# The effects of bone marrow stem and progenitor cell seeding on urinary bladder tissue regeneration

**DOI:** 10.1038/s41598-021-81939-5

**Published:** 2021-01-27

**Authors:** Matthew I. Bury, Natalie J. Fuller, Renea M. Sturm, Rebecca R. Rabizadeh, Bonnie G. Nolan, Milica Barac, Sonia S. Edassery, Yvonne Y. Chan, Arun K. Sharma

**Affiliations:** 1 Division of Pediatric Urology, Ann & Robert H. Lurie Children’s Hospital, 155 East Chicago Ave., Chicago, IL 60611 USA; 2 Department of Biomedical Engineering, Northwestern University, 2145 Sheridan Road, Evanston, IL 60208 USA; 3 Northwestern University, Simpson Querrey Biomedical Research Institute, 303 East Superior St., Chicago, IL 60611 USA; 4 Stanley Manne Children’s Research Institute, 303 East Superior St., Chicago, IL 60611 USA; 5 Department of Urology, Northwestern University Feinberg School of Medicine, 676 North St. Clair, Chicago, IL 60611 USA; 6 Center for Advanced Regenerative Engineering, Northwestern University, 633 Clark St., Evanston, IL 60208 USA

**Keywords:** Haematopoietic stem cells, Mesenchymal stem cells, Urogenital diseases, Stem cells, Diseases

## Abstract

Complications associated with urinary bladder augmentation provide the motivation to delineate alternative bladder tissue regenerative engineering strategies. We describe the results of varying the proportion of bone marrow (BM) mesenchymal stem cells (MSCs) to CD34 + hematopoietic stem/progenitor cells (HSPCs) co-seeded onto synthetic POC [poly(1,8 octamethylene citrate)] or small intestinal submucosa (SIS) scaffolds and their contribution to bladder tissue regeneration. Human BM MSCs and CD34 + HSPCs were co-seeded onto POC or SIS scaffolds at cell ratios of 50 K CD34 + HSPCs/15 K MSCs (CD34-50/MSC15); 50 K CD34 + HSPCs/30 K MSCs (CD34-50/MSC30); 100 K CD34 + HSPCs/15 K MSCs (CD34-100/MSC15); and 100 K CD34 + HSPCs/30 K MSCs (CD34-100/MSC30), in male (M/POC; M/SIS; n = 6/cell seeded scaffold) and female (F/POC; F/SIS; n = 6/cell seeded scaffold) nude rats (n = 96 total animals). Explanted scaffold/composite augmented bladder tissue underwent quantitative morphometrics following histological staining taking into account the presence (S+) or absence (S−) of bladder stones. Urodynamic studies were also performed. Regarding regenerated tissue vascularization, an upward shift was detected for some higher seeded density groups including the CD34-100/MSC30 groups [F/POC S− CD34-100/MSC30 230.5 ± 12.4; F/POC S+ CD34-100/MSC30 245.6 ± 23.4; F/SIS S+ CD34-100/MSC30 278.1; F/SIS S− CD34-100/MSC30 187.4 ± 8.1; (vessels/mm^2^)]. Similarly, a potential trend toward increased levels of percent muscle (≥ 45% muscle) with higher seeding densities was observed for F/POC S− [CD34-50/MSC30 48.8 ± 2.2; CD34-100/MSC15 53.9 ± 2.8; CD34-100/MSC30 50.7 ± 1.7] and for F/SIS S− [CD34-100/MSC15 47.1 ± 1.6; CD34-100/MSC30 51.2 ± 2.3]. As a potential trend, higher MSC/CD34 + HSPCs cell seeding densities generally tended to increase levels of tissue vascularization and aided with bladder muscle growth. Data suggest that increasing cell seeding density has the potential to enhance bladder tissue regeneration in our model.

## Introduction

Effected urinary bladder tissue secondary to disease or trauma may result in the physiological decompensation of the bladder^1,2^. Bladder decompensation is the inability of bladder smooth muscle cells (SMCs) to properly metabolize extrinsic calcium accompanied by downstream neuronal deficits^3^. This dysfunction in metabolism can lead dysregulated detrusor muscle contraction and subsequent decompensation. This is evident in individuals diagnosed with bladder outlet obstruction (BOO) where BOO can lead to an increased prevalence of bladder stone formation, urinary tract infections and ultimately renal failure, if not properly treated. This is also evidenced in several patient populations such as those afflicted with progressively deteriorating idiopathic interstitial cystitis, neurogenic bladder, radiation cystitis following bladder cancer treatment, and military personnel subjected to battlefield injuries, including exposure to improvised explosive devices and resulting pelvic injury^4–7^. Combat military personnel are three times more likely than civilians to encounter open pelvic fractures leading to perineal and pelvic tissue damage. In these instances, end-stage bladders typically undergo ileal conduit urinary diversion or bladder augmentation enterocystoplasty surgeries in order to repair or reconstruct dysfunctional bladder tissue. These procedures, in part, are typically undertaken to create an environment with the intent to increase bladder capacity and compliance and re-establish low pressure storage in order to avoid upper urinary tract deterioration^7–10^. However, each of these procedures are accompanied by numerous short- and long-term side effects that can increase patient morbidity and negatively impact health related quality of life outcomes^11–13^.

Convergent regenerative engineering approaches have been undertaken in an attempt to create bladder tissue that can reliably mimic the normal traits of native tissue in structure and physiological function^14–17^. The ultimate goal of these undertakings is the directed translational application for failing human bladder tissue in multiple clinical settings. Research endeavors such as these have led to two independent but similarly structured clinical trials^18,19^. Data from these trials yielded invaluable clinical information but ultimately, and rightfully so, demonstrated a critical need to improve approach and implementation of grafted cell/scaffold composites with an emphasis on scaffold material and cell sourcing. To date, the use of synthetic polymerics combined with autologous bladder SMCs and urothelial cells (UC) has not been able to reliably and convincingly demonstrate the regeneration of physiologically relevant bladder tissue in the clinical setting. This is especially pertinent with regards to whole bladder tissue vascularization and functional bladder smooth muscle regeneration. As an example, a recent clinical trial that utilizes amniotic membrane is attempting to treat fistula formation that often develops following hypospadias repair and bladder neck transections^20^. Unfortunately there are several drawbacks when utilizing human amniotic membranes including obtaining amniotic tissue samples that are reproducible with regards to protein expression and mechanical characteristics, and a lack of standardization when dealing with tissue preparation^21^. Thus, scaffold choice must be carefully considered. Hence, there still exists a clinical need to explore and develop methodologies that can be utilized to overcome hurdles in current bladder tissue regenerative engineering efforts.

Ongoing strategies have also been used to reconstruct bladder tissue that utilize pliable scaffolds that mimic the active and passive biaxial mechanical properties of the bladder in combination with previously well characterized stem cell populations for tissue regeneration^22–27^. Recently, bone marrow (BM) derived cells have demonstrated their utility in a bladder regenerative engineering setting^28^. In a proof-of-principle study, elastic POC [poly(1,8 octamethylene citrate)] scaffolds co-seeded with human spina bifida derived BM mesenchymal stem cells (MSCs) and donor matched CD34 + hematopoietic stem/progenitor cells (HSPCs) have been shown to act synergistically promoting by various facets of bladder tissue regeneration in a nude rat bladder augmentation model. It should be noted that donor/recipient mismatched CD34 + HSPCs used for bladder augmentation would require some level of immunosuppression in clinical settings so that rejection of the graft or subsequent graft versus host disease reactions would not occur. In an ideal setting, one could harvest the CD34 + HSPCs and expand them ex vivo prior to any surgical procedure. In this scenario, ample numbers of CD34 + HSPCs can be obtained, characterized, and subsequently utilized in vivo. Finally, data from that study demonstrated significant whole-graft tissue vascularization, peripheral nerve regeneration, and robust smooth muscle and urothelium regeneration^28^. In these studies, however, the proportion of MSCs to CD34 + HSPCs was held static at the time of scaffold seeding. Within the context of this study, we wished to determine whether bladder tissue regeneration could be positively influenced by varying the ratios of MSCs to CD34 + HSPCs on POC scaffolds within a nude rat bladder augmentation model by making intra-scaffold anatomic and bladder physiological comparisons.

## Materials and methods

### POC [poly (1,8 octamethylene citrate)] scaffold synthesis

POC scaffold synthesis was undertaken as previously described in detail^29^. Briefly, equimolar amounts of citric acid and 1,8 octanediol (Sigma Aldrich, St. Louis, MO) were combined then melted at 160–165 °C with stirring followed by a reduction in temperature to 140 °C for 25 min for pre-polymer production. The pre-polymer was dissolved in 100% ethanol to create a 30% w/v solution. This solution was distributed into a flat glass dish and polymerized for 7 days at 55 °C in air. POC scaffolds were removed from their glass molds and placed in DMEM (Lonza, Inc. Walkersville, MD) to remove any unreacted monomer. Media was changed every 6 h for 24 h. The dimensions of the POC scaffold used in vivo were 0.5 cm length × 0.5 cm width × 0.05 cm height.

### Scaffold cell seeding

Human bone marrow MSCs (Lonza Inc.) were seeded onto the serosal side of porcine small intestinal submucosa scaffolds [(SIS; Cook Biotech, West Lafayette, IN; (0.5 cm length × 0.5 cm width × 0.01 cm height)] or the aforementioned POC scaffolds under standard culture conditions (37 °C, 5% CO_2_ in air). Prior to cell seeding, scaffolds were primed in mesenchymal stem cell growth medium (MSCGM, Lonza Inc.) for two days under the same culture conditions. MSCs were allowed to attach and proliferate onto each respective scaffold for 7–10 days until approximately 80% confluent. Following MSC scaffold culturing, MSCGM culture media was removed and exchanged with a 1:1 mixture of MSCGM and Hematopoietic Progenitor Growth Medium (Lonza, Inc.). Human bone marrow CD34 + HSPCs (Lonza, Inc.) were then co-seeded onto the serosal side of the scaffolds and allowed to attach overnight prior to bladder augmentation surgery the following day. SIS and POC scaffolds were independently seeded at the following cell ratios: (1) 50 K CD34 + HSPC/15 K MSC (CD34-50/MSC15), (2) 50 K CD34 + HSPC/30 K MSC (CD34-50/MSC30), (3) 100 K CD34 + HSPC/15 K MSC (CD34-100/MSC15), (4) 100 K CD34 + HSPC/30 K MSC (CD34-100/MSC30). K = 1,000. Cell viability was determined by NucBlue live cell stain (Thermo Fisher Scientific, Waltham, MA) prior to bladder augmentation surgeries. Unseeded POC and SIS scaffolds were not utilized for augmentation studies as they do not promote bladder tissue regeneration as previously demonstrated^17,29^.

### In vivo bladder augmentation studies

Athymic nude rats (male and female weighing ~ 200 g; 10 weeks of age; Charles River Laboratories, Wilmington, MA) underwent bladder augmentation as previously described^17^. Summarily, rats were anesthetized with inhalation of 2% isoflurane followed by buprenex (1 mg/kg) to mitigate pain during and after surgery. A 1.0 cm midline vertical skin incision was created to expose the abdominal fascia and muscles with subsequent positive identification of the urinary bladder. A 50–70% supratrigonal cystectomy was performed from anterior to posterior part of the bladder. The newly cystectomized bladder defect was then independently augmented with either SIS or POC scaffolds containing MSC and CD34 + HSPCs in the following ratios: (1) CD34-50/MSC15, (2) CD34-50/MSC30, (3) CD34-100/MSC15), (4) CD34-100/MSC30 in male and female rats (n = 6 for each sex at the onset of the study; n = 96 total animals utilized. 11 animals did not survive the entirety of the study due to bladder leakage, stone formation/retention, or idiopathic origins). The bladder was closed with 7–0 polyglactin suture in a watertight manner and subsequently sutured with surrounding omentum, followed by the closure of the abdominal wall with 4–0 chromic running suture and the skin re-approximated with 9 mm autoclips. Athymic nude rats were utilized for this study to allow for the engraftment and subsequent physiological response of human cells and to examine the innate immune response to inflammation without influence of the adaptive immune response. All animal studies were performed in accordance with ARRIVE guidelines and those set forth and approved by the Northwestern University Institutional Animal Care and Use Committee (IACUC).

### Tissue specimen processing/staining

Whole bladders and kidneys were removed from augmented animals following euthanasia 10 weeks post-augmentation. Specimens were fixed in a 10% buffered formalin phosphate, dehydrated with exchanges of graduated ethanol, embedded in paraffin, and sectioned onto glass slides at a 5 µm thickness. Slides were deparaffinized with xylenes, graduated ethanol washes, and deionized water. This was followed by an established staining protocol for Masson’s trichrome^29^. Slides were also independently stained with H&E using an established protocol^30^. Slides were consecutively deparaffinized with xylenes, dehydrated with ethanol changes and re-hydrated with deionized water. After these steps, slides were stained with H&E, followed by washes with gradient ethanol solutions. Following air drying, a coverslip was placed over the specimen sample and secured with Permaslip (Alban Scientific Inc.). A total of 85 bladders underwent tissue analysis from all experimental groups.

### Blood vessel quantification in areas of bladder tissue regeneration

Digitized sample images that were initially stained with Masson’s trichrome were opened with Adobe Photoshop CS3 (Adobe Systems Inc.). These samples were further characterized with a Nikon Eclipse 50i Microscope (Nikon Inc., Melville, NY) and Spot Advanced Imaging Software (Diagnostic Instruments, Sterling Heights, MI) as previously described by our laboratory using established methods^29^. Utilizing the pen tool in Spot, vessel numbers were quantified based upon n = 10 images per graft in bladder tissue regenerated areas. Individual vessels were manually selected and quantified using the image histogram tool to acquire pixel density for each vessel. Data is represented as mean number of vessels/mm^2^ (means ± SE).

### Bladder stone evaluation and kidney evaluation

The presence/absence of bladder stones was noted visually immediately following euthanasia. Stones present in the bladder were manually counted and subsequently a total stone burden (TSB; volumetric sum of stones) was derived for each S+ (stone positive) animal. Kidney cross sections were stained with H&E and digitally imaged utilizing a Nikon Eclipse 50i Microscope for S+ and S− (stone negative) animals. 4X images were opened and using the calibrated area tool on Spot Advanced Imaging Software the area of renal pelvic dilation (RPD) was identified and measured (in mm^2^). 3 images/sample. Degrees of kidney hydronephrosis were subjectively determined by a trained urologist (YYC).

### Bladder tissue muscle quantification

With regards to bladder tissue muscle quantification, an established protocol was utilized by our laboratory to determine collagen expression^28,29^. Masson’s trichrome stained samples were digitally quantified for collagen expression using a Nikon Eclipse 50i Microscope and Spot Advanced Imaging Software. Sample images (1600 pixels-1200 pixels, bit depth 24) were opened with Adobe Photoshop CS3 (Adobe Systems Inc., San Jose, CA). The contrast of red pixels from blue pixels was enhanced by a two-fold elevation of magenta levels followed by a two-fold depression of cyan levels in the red and magenta spectra. This contrast was further improved by a two-fold elevation of cyan levels followed by a two-fold depression of magenta levels in the cyan and blue spectra. The selection color range tool with a fuzziness level of 125% was then used to digitally select the red or blue pixels of the entire image. Selected pixels were subsequently quantified using the image histogram tool and a muscle to collagen ratio was calculated from these values as described by our laboratory^28,29^. In instances where UCs, red blood cells, debris, and the SIS or POC scaffold were present, images were edited to remove these structures to preserve a more accurate extrapolation of the muscle content from the red/blue^28,29^. Areas of regenerated tissue were subjected to an average of ten random microscopic fields to determine muscle levels at 10 weeks post-augmentation. Data was based upon ten images per animal for each group at the 10 week timepoint.

### Urodynamic studies and bladder capacity evaluation

Urodynamic assessment and bladder capacity measurements were performed prior to bladder augmentation and euthanization as described^31^. The bladders of anesthetized athymic nude rats were exposed through a lower abdominal incision. A 20 gauge cannula (Becton Dickinson, Franklin Lakes, NJ) was inserted into the bladder dome and connected to the Pump 11 Elite Syringe Pump (Harvard Apparatus, Holliston, MA) and to a physiological pressure transducer (SP844, MEMSCAP). The pressure transducer was connected to a bridge amplifier (Model FE221; AD Instruments, Colorado Springs, CO), which record and plotted continuous readings of the transvesical pressures using LabChart 7.3 Software (AD Instruments). Prior to filling, the bladder was manually decompressed to ensure that it was empty. The bladder was then filled at 150 µl/min. Bladder capacity was estimated by the product of flow rate and time to urethral leakage. Voiding pressure was the maximum pressure during the terminal contraction. Intravesical urodynamic measurements could not be performed at other timepoints of this study due to the highly invasive nature of the testing procedure.

### Statistical analysis

Occurrence of bladder stones in 33/85 animals across constructs required seeding density groups to be broken into S+ and S− subsets, reducing the expected n for some groups. For each of the four constructs (F/POC, F/SIS, M/POC, M/SIS), differences between the S− CD34-50/MSC15 group and the three increased cell seeding density groups (S− CD34-50/MSC30, S− CD34-100/MSC15 and S− CD34-100/MSC30), and differences between the S+ CD34-50/MSC15 group and the three increased cell seeding density groups (S+ CD34-50/MSC30, S+ CD34-100/MSC15 and S+ CD34-100/MSC30) were determined using ANOVA with Dunnett’s post-hoc test. Not all construct subsets retained enough animals for analysis. In instances of similar S+ and S− subset trends for a construct, constructs were further assessed without subsets (any significant differences described include details of the specific comparison). P < 0.05 was considered statistically significant. Analyses were performed with SAS 9.4 software (SAS Institute)^29^.

### Ethical standards

All animal studies were performed in accordance with guidelines set forth and approved by the Institutional Animal Care and Use Committee (IACUC) at Northwestern University.

## Results

### Blood vessel quantification following scaffold augmentation

**Figure 1:**
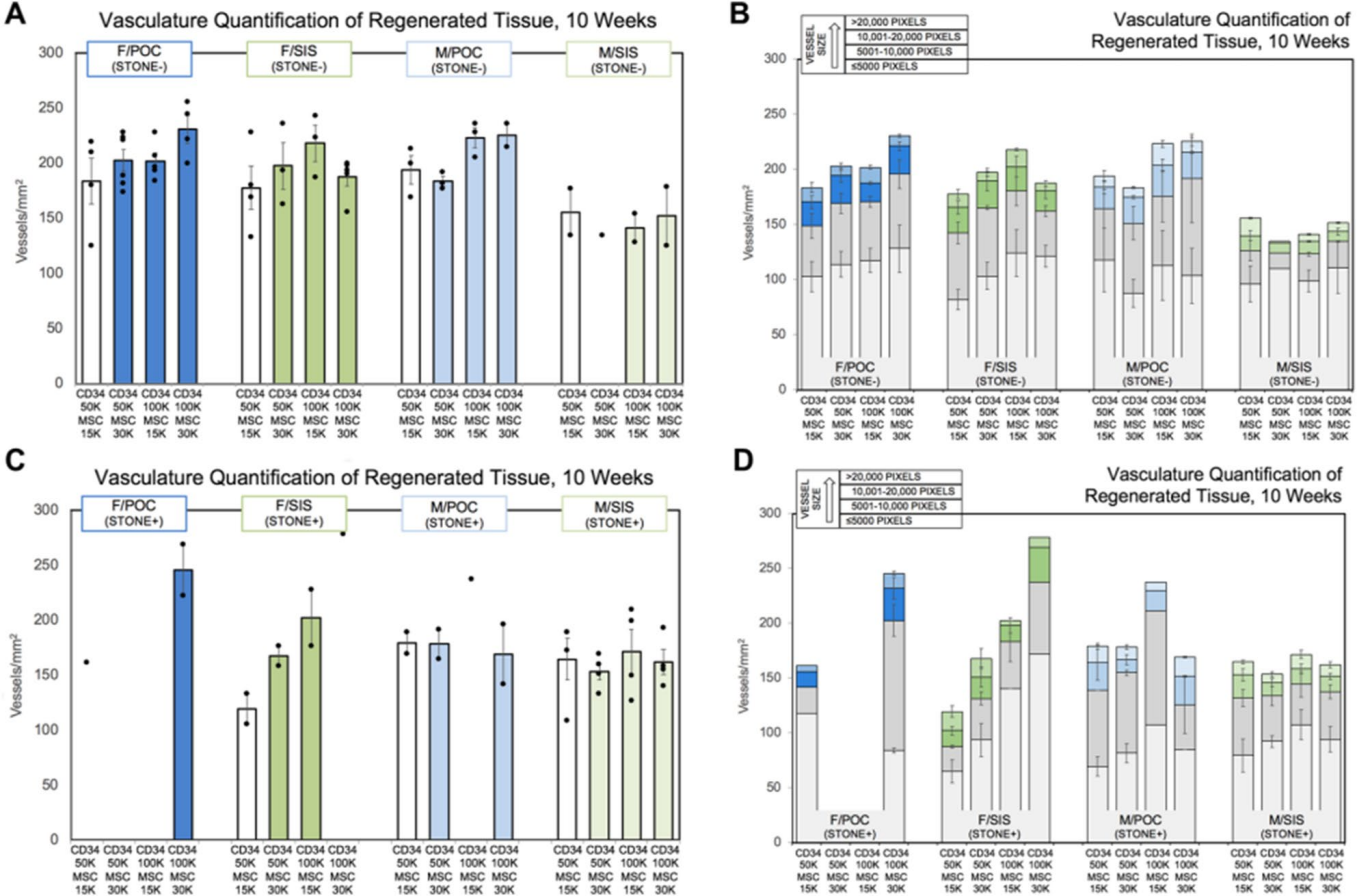
Analysis of vasculature in regenerated female and male rat bladder tissue sections10 weeks post-augmentation. Average vasculature per group is measured in vessels/mm^2^ for (**A**, **B**) stone negative (S−) and (**C**, **D**) stone positive (S+) rats augmented with either a POC or SIS scaffold seeded at a specific cellular concentration with MSC and CD34 + HSPC at varying ratios (n = 4–6 before subsets). All female graft groups (S− and S+) and M/POC (S−) demonstrate a possible trend towards increased vasculature at greater seeding densities. In assessment without subsets, the increase for F/POC (S− and S+ animals) was significant [CD34-50/MSC15 179.2 ± 17.0 vs CD34-100/MSC30 235.5 ± 10.4 (p < 0.01)]. B/D) Vessels are shown grouped by size. Trends toward increases in some size groups were observed (F/POC S− and S+, and M/POC S− 5001–10,000 size; F/SIS S− and S+  ≤ 5000 size). In assessment without subsets, the increases for F/POC (S− and S+ animals) and F/SIS (S− and S+ animals) were significant [F/POC CD34-50/MSC15 41.7 ± 9.9 vs CD34-100/MSC30 84.6 ± 14.0 (p < 0.05); F/SIS CD34-50/MSC15 76.3 ± 7.4 vs CD34-100/MSC15 130.9 ± 12.2 (p < 0.01) and CD34-100/MSC30 129.7 ± 11.7 (p < 0.01)]. Error bars represent ± SE.

Levels of vasculature in regenerated tissue were possibly affected by increased seeding density in the F/POC (S− and S+), F/SIS (S− and S+), and M/POC (S−) constructs, as an upward shift in range of vessels/mm^2^ was detected for some higher seeding density groups (**F/POC S−** CD34-50/MSC15 183.6 ± 21.1, range 125.4–219.1; CD34-50/MSC30 202.8 ± 9.8, range 173.8–228.2; CD34-100/MSC15 201.9 ± 7.5, range 184.4–228.2; CD34-100/MSC30 230.5 ± 12.4, range 199.5–255.4 and **F/POC S+ **CD34-50/MSC15 161.7; CD34-100/MSC30 245.6 ± 23.4, range 222.2–269.0; **F/SIS S−** CD34-50/MSC15 177.6 ± 19.6, range 133.0–228.2; CD34-50/MSC30 197.5 ± 21.0, range 163.2–235.8; CD34-100/MSC15 218.1 ± 16.4, range 187.4–243.3; CD34-100/MSC30 187.4 ± 8.1, range 155.7–199.5 and **F/SIS S+ **CD34-50/MSC15 119.4 ± 13.6, range 105.8–133.0; CD34-50/MSC30 167.8 ± 9.1, range 158.7–176.8; CD34-100/MSC15 202.5 ± 25.7, range 176.8–228.2; CD34-100/MSC30 278.1; **M/POC S−** CD34-50/MSC15 194.0 ± 13.0, range 169.3–213.1; CD34-50/MSC30 183.4 ± 4.5, range 176.8–191.9; CD34-100/MSC15 223.2 ± 9.1, range 205.5–235.8; CD34-100/MSC30 225.2 ± 10.6, range 214.6–235.8) (Fig. 1A,C). This was not observed for the M/SIS construct (Fig. 1A,C). Presence of stones did not appear to impact the number of vessels/mm^2^ in a consistent manner.

Differences in vasculature were further examined through assessment of blood vessel size in regenerated tissue (as determined by pixel count group designation: ≤ 5000; 5001–10,000; 10,001–20,000; > 20,000). For F/POC S− and S+, a trend toward upward shifts for the 5001–10,000 size at higher seeding density was observed (Fig. 1B,D). For F/SIS S− and S+, the upward shift trend for higher seeding density was apparent for the ≤ 5000 size, and for M/POC S−, the upward shift trend was at the 5001–10,000 size (Fig. 1B,D). Upward shifts were not detected in M/SIS S−, and this construct differed from the others with a reduced range of vessels at the 5001–10,000 size (Fig. 1B). In constructs with similar S+ and S− subset trends (F/POC, F/SIS), the increase in total vessels/mm^2^ (F/POC) or vessels/mm^2^ of a particular size (F/POC, F/SIS) observed with higher seeding densities was statistically significant [assessed without S+/S− subsets; F/POC (S+ and S− animals) total vessels/mm^2^ CD34-50/MSC15 179.2 ± 17.0 vs CD34-100/MSC30 235.5 ± 10.4 (p < 0.01) and vessels/mm^2^, 5001–10,000 size, CD34-50/MSC15 41.7 ± 9.9 vs CD34-100/MSC30 84.6 ± 14.0 (p < 0.05); F/SIS (S+ and S− animals) vessels/mm^2^, ≤ 5000 size, CD34-50/MSC15 76.3 ± 7.4 vs CD34-100/MSC15 130.9 ± 12.2 (p < 0.01) and CD34-100/MSC30 129.7 ± 11.7 (p < 0.01)]. Aside from these comparisons, differences between CD34-50/MSC15 and greater cell seeding density groups were not determined to be statistically significant for any construct. It should be noted that 11 animals did not survive the entirety of the study as previously described.

### Bladder stone and kidney evaluation following bladder augmentation

**Figure 2:**
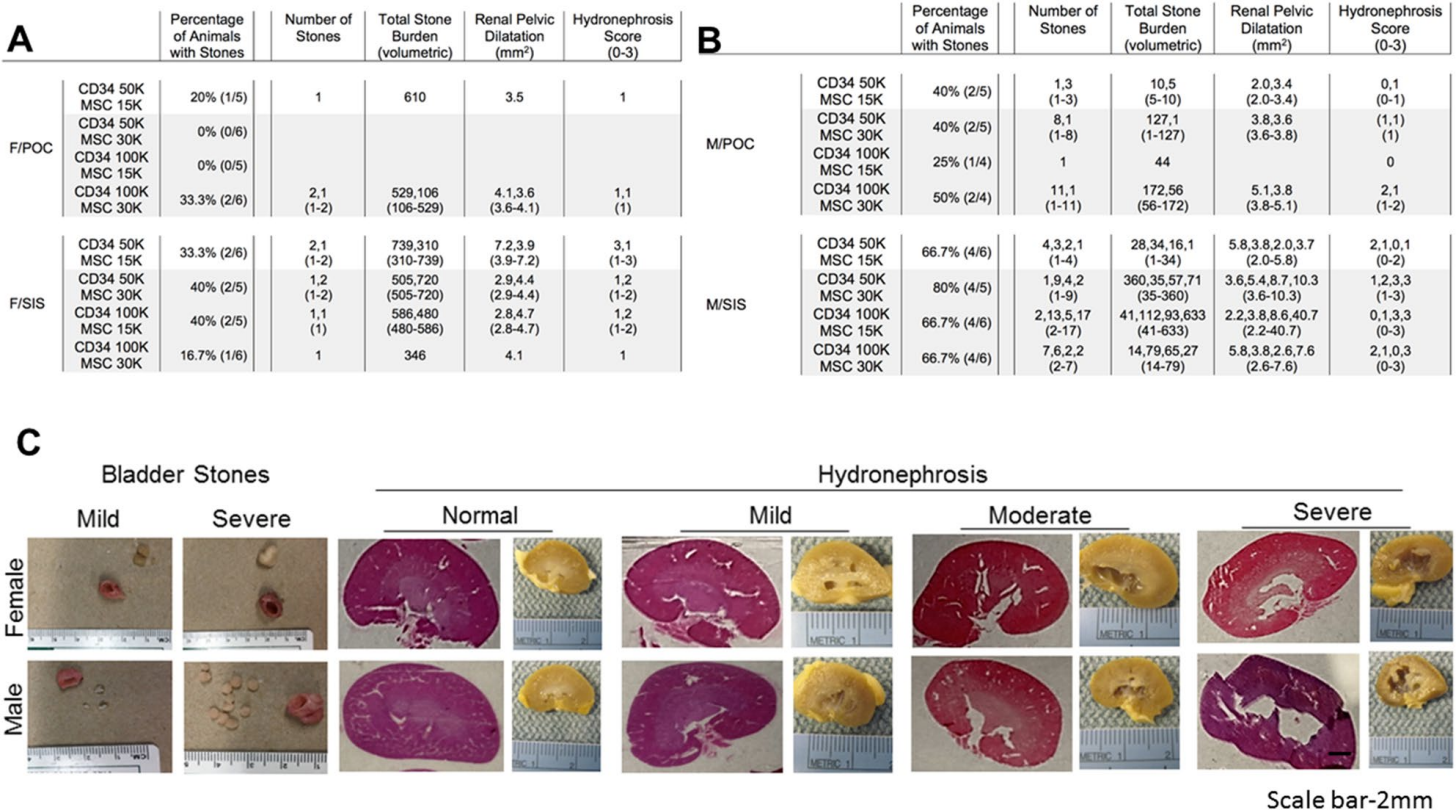
Characterization of the condition of 10-week post-augmentation rat bladders and kidneys. (**A**, **B**) Tables show percentage of rats with bladder stones, number of stones, total stone burden (volumetric), renal pelvic dilatation (mm^2^) and a hydronephrosis score resulting from varying ratios in the cell concentration of MSC and CD34 + HSPCs seeded on POC/SIS scaffolds (n = 4–6 before subsets). Female groups displayed a lesser percentage of stone positive animals and range in number of stones (22.7% and 1–2 stones) when compared to male groups (56.1% and 1–17). On average, male rats produced a greater number of smaller sized stones than female rats. (**C**) Images and H&E stains demonstrate the range in severity of bladder stone formation and hydronephrosis, between female and male augmented rats (1x). Scale bar: 2 mm.

Bladder stones developed in 10/44 (22.7%) female animals [F/POC 3/22 (13.6%), F/SIS 7/22 (31.8%)], with a similar number of stones/animal (1–2) across groups (Fig. 2A). The TSB range was 106–610 for F/POC and 310–739 for F/SIS, with an elevated TSB range in F/SIS for two stones vs one (720–739 vs 310–586) (Fig. 2A). When compared to a subset of animals without stones (S−; n = 3–5 per composite graft), the renal pelvic dilatation (RPD; maximum width of renal pelvis) range for the S+ animals reflected a trend toward higher values [F/POC S+ 3.5–4.1 vs S- 1.8–2.5; F/SIS S+ 2.8–7.2 vs S− 1.9–3.6]. The hydronephrosis (H) score [0–3, denoting normal (0), mild (1), moderate (2) or severe (3)] was consistent across the three F/POC S+ animals at 1 (mild), and ranged from 1 to 3 (mild to severe) for F/SIS S+ animals. For the F/SIS animals, higher H scores tended to be associated with higher RPD values (H = 1, RPD 2.8–4.1; H = 2, RPD 4.4–4.7; H = 3, RPD 7.2). The maximum H and RPD values (3; 7.2) were associated with the highest TSB (739), but at lower H score gradations and RPD values, no clear relationship with TSB was evident (Fig. 2A).

In male composite grafts, stones were detected in 23/41 (56.1%) animals [M/POC 7/18 (38.9%), M/SIS 16/23 (69.6%)], a greater percentage than observed in female groups (Fig. 2B). In comparison to female S+ animals, an expanded range of stones/animal was observed (M/POC 1–11 and M/SIS 1–17 vs F/POC and F/SIS 1–2), with a higher number of stones and lower TSB in most instances. While TSB > 100 was observed for all ten female S+ animals (TSB range 106–739), only 2/7 (M/POC) and 3/16 (M/SIS) male S+ animals reached that level of TSB. For the M/POC composite grafts (TSB range 1–172), only animals with the highest numbers of stones (8, 11) had a TSB > 100 (127, 172). In M/SIS S+ animals (TSB range 1–633), TSB was < 100 for 13/16 (stone number 1–9) and > 100 for 13/16, with high stone numbers for two of the three > 100 (stone number 1, 13, 17) (Fig. 2B). The RPD range for S+ animals indicated a trend toward higher maximum values when compared to the range for a subset of S− animals [M/POC S+ 2.0–5.1 vs S− 2.2–2.6; M/SIS S+ 2.0–40.7 vs S− 2.3–3.7] (Fig. 2B). H scores for the M/POC group were relatively low (H = 0–1 6/7 animals; H = 2 1/7 animals), while the M/SIS group included more animals with higher H scores (H = 0–1 8/16 animals; H = 2 3/16 animals; H = 3 5/16 animals). As observed in the F/SIS group, maximum H and RPD values were associated with the highest TSB, but this relationship was not clearly observed at lower H score gradations and RPD values (Fig. 2B). Images in Fig. 2C depict mild and severe stone formation found in male and female augmented animals. This was accompanied by different degrees of hydronephrosis (ranging from normal to mild to moderate to severe) that can be observed in H&E stained kidney samples (Fig. 2C).

### Muscle quantification

**Figure 3:**
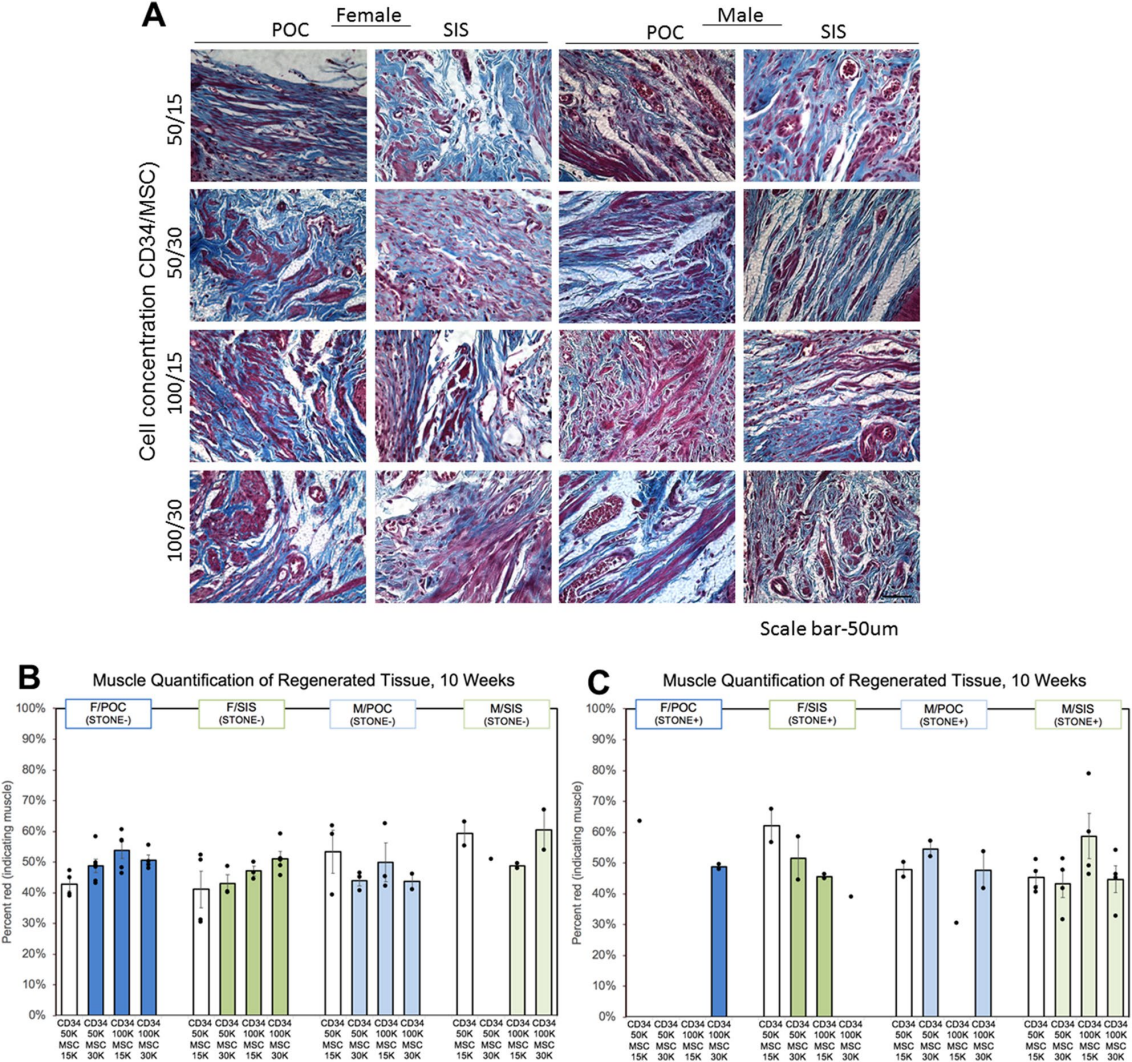
Masson’s trichrome stains of 10-week regenerated rat bladder tissue following augmentation (40 ×). (**A**) Images of female and male bladder tissue sections show the distribution of muscle and collagen in the regenerated tissue that is representative of the animals in that graft group (n = 4–6 before subsets; 10 images per animal). Analysis of the regenerated tissue examines muscle tissue area as a percent of total regenerated bladder tissue for each group further divided into (**B**) stone negative (S−) and (**C**) stone positive (S+) rats. Further analysis shows a potential trend towards increased percent muscle area at higher seeding densities. In S− F/POC and F/SIS groups, a greater fraction of animals in each seeding group achieved ≥ 45% muscle as seeding densities increased, however, group differences were generally not statistically significant. A comparison of seeding groups CD34-50/MSC15 and CD34-100/MSC15 (F/POC S−) generated the only statistically significant group difference (p < 0.05). Scale bar: 50 μm. Error bars represent ± SE.

Masson’s trichrome staining demonstrates typical tissue regeneration in female and male POC and SIS constructs (Fig. 3A). In regenerated tissue, a possible trend toward increased levels of percent muscle with higher seeding densities was observed for F/POC and F/SIS S− subsets. For F/POC, only 1/4 animals in the lowest seeding density group (CD34-50/MSC15) achieved ≥ 45% muscle (42.7 ± 2.0, range 39.0–47.2), vs 4/6, 5/5 and 4/4 animals in the higher seeding density groups (CD34-50/MSC30 48.8 ± 2.2, range 43.1–58.4; CD34-100/MSC15 53.9 ± 2.8, range 46.4–60.7; CD34-100/MSC30 50.7 ± 1.7, range 48.0–55.5). Similarly, for F/SIS, only 2/4 and 1/3 animals reached ≥ 45% muscle at the lowest seeding densities (CD34-50/MSC15 41.2 ± 6.0, range 30.5–52.3; CD34-50/MSC30 43.1 ± 2.9, range 40.1–48.8), and two of the animals in the CD34-50/MSC15 group measured < 35% muscle. At the higher seeding densities, 2/3 and 5/5 F/SIS animals were at the ≥ 45% muscle level (CD34-100/MSC15 47.1 ± 1.6, range 44.5–50.3; CD34-100/MSC30 51.2 ± 2.3, range 45.7–59.3). This trend was not evident in F/POC and F/SIS S+ subsets, as higher values were calculated for the lowest seeding density groups. Of note, the most extreme values were detected in the F/SIS lowest seeding density group (CD34-50/MSC15: 2/4 animals < 35% in the S− subset; 1/2 animals > 65% in the S+ subset) (Figs. 3B and 3C).

No clear effect of seeding density on percent muscle was detected for M/POC and M/SIS, for either S− or S+ subsets. Across M/POC S− groups, similar minimum values and a similar percentage of animals achieving levels ≥ 45% muscle (2/3, 2/3, 2/3, 1/2; 50–66.7%) were observed, with varying maximum levels (CD34-50/MSC30 53.4 ± 7.1, range 39.3–61.8; CD34-50/MSC30 44.0 ± 1.8, range 40.6–46.4; CD34-100/MSC15 49.9 ± 6.3, range 42.2–62.4; CD34-100/MSC30 43.7 ± 2.6, range 41.1–46.2). M/POC S+ subsets did not show an evident relationship between seeding density and percent muscle, or a consistent impact of stone presence on percent muscle (percentage of animals achieving levels ≥ 45% 0–100% across groups; 2/2, 2/2, 0/1, 1/2). All M/SIS S− animals reached ≥ 45% muscle (2/2, 1/1, 2/2, 2/2; CD34-50/MSC30 59.2 ± 3.9, range 55.4–63.1; CD34-50/MSC30 51.1; CD34-100/MSC15 48.9 ± 0.8, range 48.0–49.7; CD34-100/MSC30 60.5 ± 6.5, range 54.0–67.0). The M/SIS S+ subsets displayed some decrease in minimum levels, leading to lower percentages of animals achieving levels ≥ 45% (50–100% across groups; 2/4, 2/4, 4/4, 3/4). M/SIS had more extreme values than other constructs (1/2 animals > 65% in the CD34-100/MSC30 S− subset; 1/4 animals < 35% in each of CD34-50/MSC30 and CD34-100/MSC30 S+ subsets, and 1/4 animals > 65% in the CD34-100/MSC15 S+ subset) (Fig. 3B,C). Apart from F/POC S- CD34-50/MSC15 vs CD34-100/MSC15 (p < 0.05), differences between CD34-50/MSC15 and greater cell seeding density groups were not determined to be statistically significant for any construct.

### Urodynamic studies

**Figure 4:**
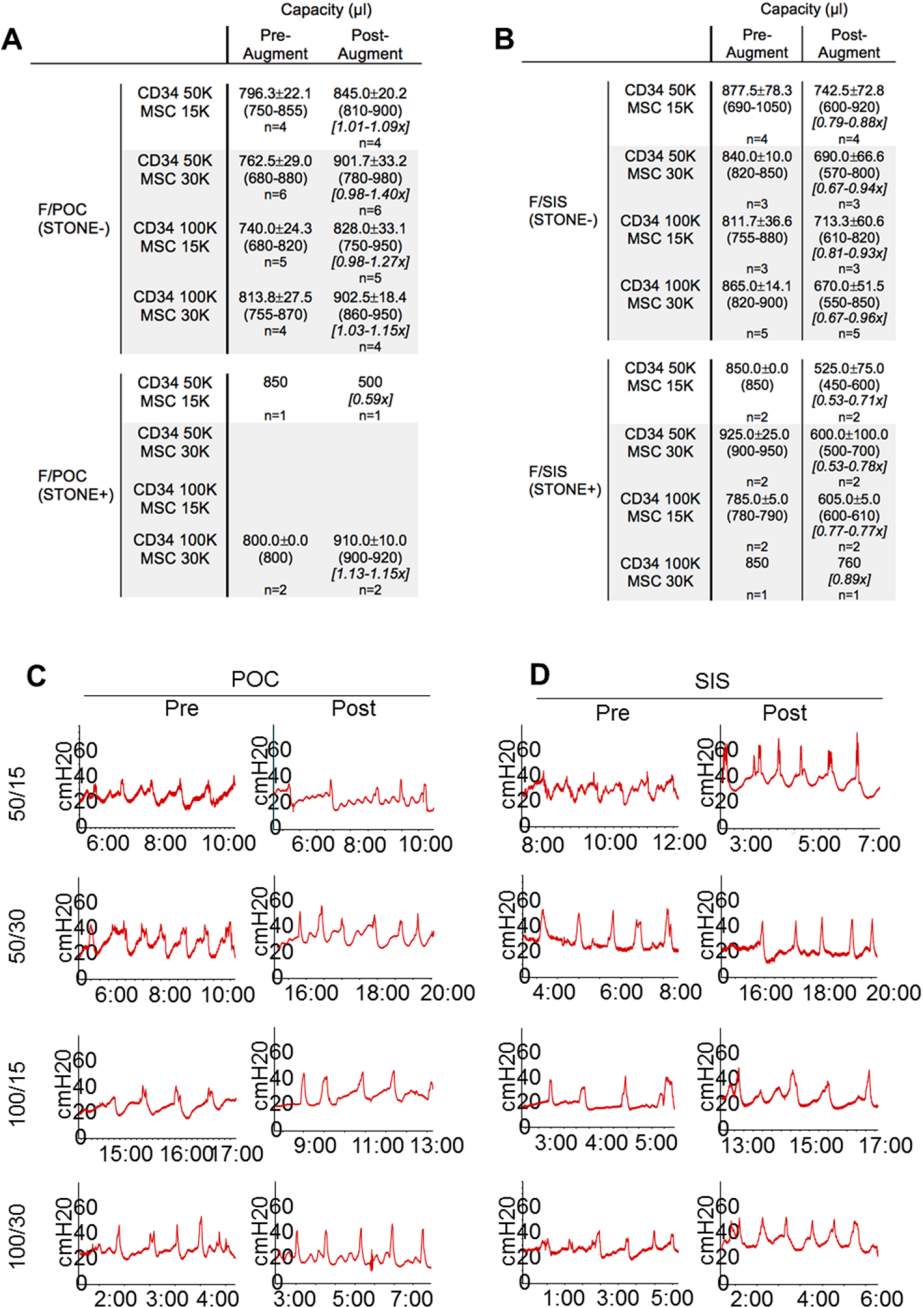
Urodynamic studies (UDS) demonstrate the physiological function of female rat bladders pre- and post-augmentation with either (**A**) POC or (**B**) SIS scaffolds seeded at a specific cellular concentration and ratio with MSC and CD34 + HSPC. Female graft groups are further divided by the absence (S−) or presence (S+) of bladder stones, which, based on our results are thought to alter the bladder’s true capacity. (**A**, **B**) Tables display the mean filling capacity (μl) and range in post-augment/pre-augment capacity ratios for each composite graft (n = 5–6 before subsets). The only female rat group to demonstrate post-augment/pre-augment ratios ≥ 0.9 at all seeding levels was F/POC (S−). (**C**, **D**) UDS readings are representative of each of the female groups and demonstrate the rise in intravesical pressure (cmH20) leading to a contraction of the bladder.

**Figure 5:**
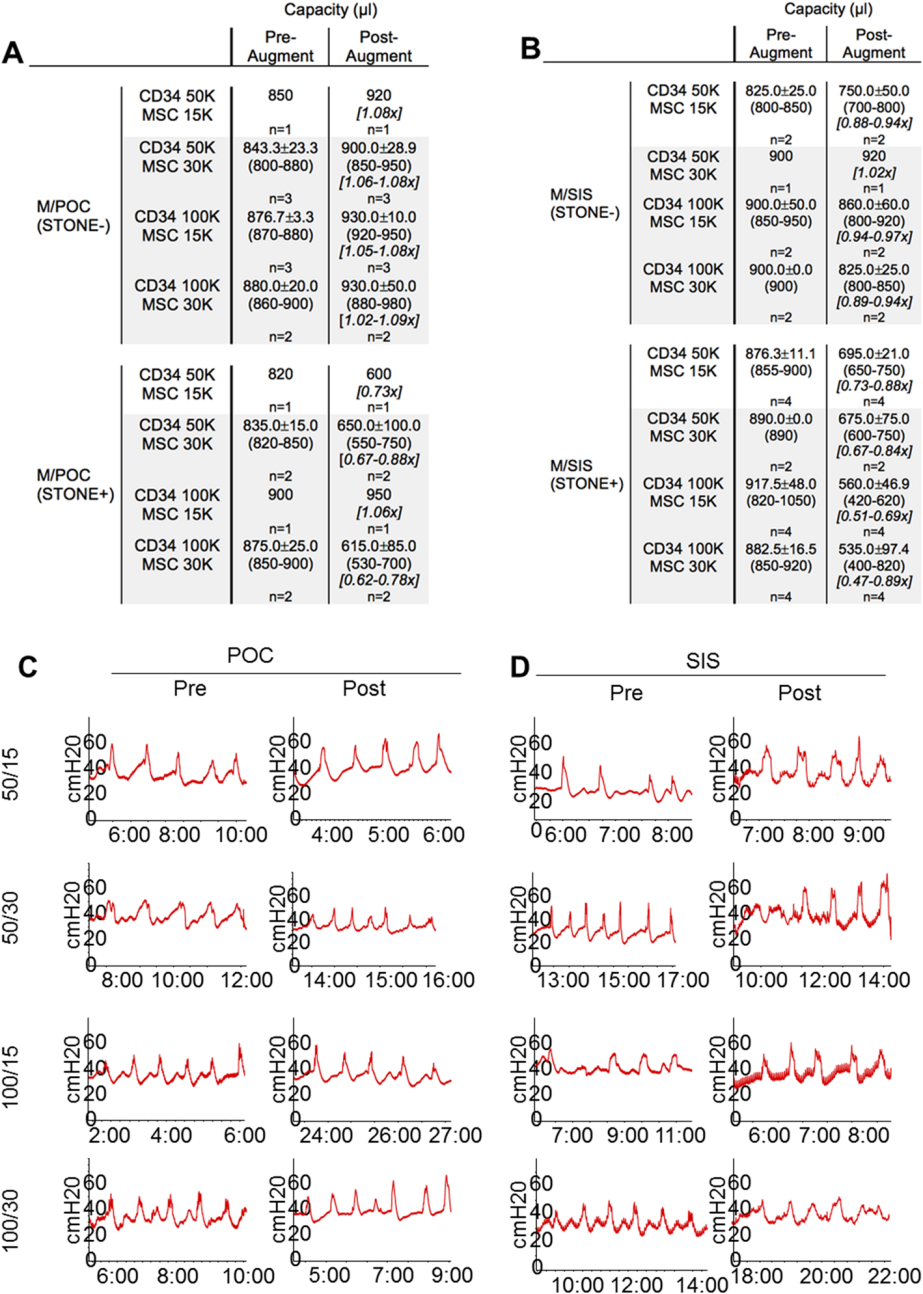
Urodynamic testing on male rat bladders pre- and post-augmentation with (**A**) POC or (**B**) SIS scaffolds seeded at a specific cellular concentration (cells/cm^2^) with MSC and CD34 + HSPC at varying ratios. Male graft groups are further divided into S− and S+ animals, separated due to the effect bladder stones may have on the actual bladder capacity. A/B) Tables display the mean filling capacity (μl) and range in post-augment/pre-augment capacity ratios of the bladders (n = 2–6 before subsets). Rats in the M/POC (S−) construct were the only group out of 4 to achieve post-augment/pre-augment ratios over 1.0 × in all seeding groups. (**C**, **D**) UDS readings are from a single group individual and are representative of the group as a whole.

Capacity was evaluated pre- and post-augmentation for all groups. For POC (S−) groups, post-augment/pre-augment capacity ratios were ≥ 0.9 for all animals across all groups (F/POC 19/19, range 0.98–1.4; M/POC 9/9, range 1.02–1.09), with 10/19 F/POC animals ≥ 1.1. Mean ratios for the four seeding density groups ranged from 1.06 to 1.19 for F/POC and from 1.06 to 1.08 for M/POC (Figs. 4A and 5A). For SIS (S−) groups, post-augment/pre-augment capacity ratios were ≥ 0.9 for 5/7 male animals but only 3/15 female animals (M/SIS range 0.88–1.02; F/SIS range 0.67–0.96). Mean ratios for the four seeding density groups ranged from 0.91 to 1.02 for M/SIS and from 0.77 to 0.88 for F/SIS (Figs. 4B and 5B). CD34 HSPC and MSC seeding densities did not appear to affect bladder capacity ratios regardless of scaffold type.

For all graft constructs, in comparison to S− subsets described above, S+ subsets demonstrated reduced numbers of animals with post-augment/pre-augment capacity ratios ≥ 0.9 (F/POC 19/19 to 2/3; M/POC 9/9 to 1/6; F/SIS 3/15 to 0/7; M/SIS 5/7 to 0/14) (Figs. 4A,B, 5A,B). F/POC was the least affected (ratios for two S+ CD34-100/ MSC30 animals were > 1.1). M/SIS was the most impacted, as none of the 14 S+ animals achieved post-augment/pre-augment capacity ratios ≥ 0.9 (ratios were ≤ 0.75 for 9/14, and < 0.50 for two of these animals). The presence of bladder stones may have altered true bladder capacity as the stones displace bladder volume. It is important to note that the male rats tended to present with an increased baseline bladder pressure of approximately 30–40 cm H_2_0 (Fig. 5C,D) as opposed to female rats that presented with lower pressures of approximately 20–35 cm H_2_0. (Fig. 4C,D). UDS in Figs. 4 and 5 represents a single animal at each timepoint but is representative of multiple animals that were used throughout the study. Different seeding densities did not result in bladder overactivity post-augmentation. Mild increase in baseline pressure was noted in the F/SIS (CD34-50/MSC15), M/POC (CD34-50/MSC 15), M/SIS (CD34-50/MSC 15), M/SIS (CD34-50/MSC30) animals post-augmentation. The clinical significance of this observation is unknown. Post-procedure images of the gross bladders that were seeded at different MSC and CD34 + HSPC concentrations are provided as Figure S1 in the Supplementary Information.

## Discussion

The rapidly evolving field of regenerative engineering offers the opportunity to provide novel and therapeutic treatment modalities for those suffering from acute and chronic bladder dysfunction and in need of bladder tissue replacement. However, overcoming the obstacles associated with urinary bladder regenerative engineering endeavors has been an arduous task. Bladder-based basic science and clinical regenerative engineering studies spanning greater than four decades have yielded disparate results. There is still no general consensus on how to successfully translate wet-lab derived concepts and results into functional applications with translatable relevance for various clinical scenarios. This is in part due to several critical factors that impose tremendous influence on overall bladder tissue regeneration. These include scaffold design and biocompatibility; animal model utility in which data can be reliably extrapolated to the human condition; and perhaps most importantly, cell sourcing to promote bladder tissue growth and development. These pivotal elements must emphatically coalesce to ultimately drive the functional success of in vivo grafted materials. The objective of this study was to assess the bladder tissue regenerative capacity of POC and SIS in the context of various bone marrow derived cell seeding densities in order to delineate a potential correlation between cell seeding density and bladder tissue regeneration and resulting bladder physiology.

Contemporary advancements in elastomer chemistry have allowed for the manufacture of synthetic and hybrid scaffolds that are biocompatible and are highly reproducible with regards to chemical and mechanical properties^17,28,29,32,33^. The utilization of synthetic matrices that have the ability to mimic the mechanical properties of the bladder is invaluable towards the re-creation of a realistic, native bladder environment. However, compared to biological scaffolds, purely synthetic polymerics typically lack the expression of functional pro-angiogenic or -vasculogenic molecules such as VEGF and bFGF that are conducive for tissue vascularization^34–37^. Pro-vascularizing growth factors (GFs) are typically added to scaffolds via chemical and functional modifications in order to exploit their tissue vascularizing effects in vivo^35–37^. In instances where modifications are not feasible, it becomes of paramount importance that the incorporation of cells into synthetic scaffolds will in part assume a role in graft vascularization and subsequent perfusion. This is especially relevant at the core of large grafts where oxygen and nutrient exchange can be poor, leading to tissue necrosis and subsequent graft failure^38^.

MSCs combined with CD34 + HSPCs actively participate in the formation of blood vessels in regenerating bladder tissue in a nude rat bladder augmentation model as previous studies have demonstrated^28^. Within those studies, human CD34 + HSPCs in the presence of SB human donor-matched MSCs were co-seeded onto synthetic POC scaffolds and demonstrated potent blood vessel formation within the entirety of the graft 4 weeks post-augmentation. An approximate 5.6 × − 7.2 × increase in the mean number of vessels was observed when comparing regenerated to native bladder tissue across all donor groups^28^. The addition of the CD34 + HSPCs also initiated peripheral nerve and substantial urothelium regeneration when compared to experimental controls animals within those studies^28^. Throughout these studies, MSCs and CD34 + HSPCs were seeded at a single density (MSCs at 1.5 × 10^4^ MSCs/cm^2^; CD34 + HSPCs at 1–2 × 10^5^/graft). As SIS has shown to retain multiple pro-angiogenic molecules^39^, it was unanticipated that SIS composites did not outperform POC counterparts with regards to tissue vascularization as POC scaffolds are devoid of any exogenously added GFs. Several studies have established the pro-angiogenic nature of SIS^39–42^. Lin and colleagues demonstrate the increased paracrine expression of specific angiogenesis-promoting growth factors in the conditioned media (CM) of MSCs that were cultured onto SIS disks in vitro^38^. Data indicated increased expression of GFs involved in angiogenesis by a 1.4-fold pentraxin 3, threefold IGFBP-1, sevenfold IL-8, twofold uPA, 14-fold HGF, and 1.8-fold VEGF when compared to CM derived from MSCs that were cultured on plastic^38^.

Surrogate angiogenic assays indirectly supported paracrine release of pro-angiogenic GFs from MSC/SIS disks and the promotion of tube formation. Lin et al. further demonstrate that CM obtained from MSC/SIS disks 24 h post-incubation and cultured with HMVEC-Cs (human microvascular endothelial cells–cardiac) in Matrigel increased length of tubes formed, number of nodes, branches, and junctions in a statistically relevant manner when compared to HMVEC-Cs treated with CM derived from MSCs cultured on plastic^38^. Furthermore, CD34 + HSPCs and CD34 + HSPC derived endothelial cells have been reported to undergo differentiation into endothelial cells and promote tissue vascularization under the influence of VEGF^42–44^. As SIS expresses VEGF at varying levels, we would have anticipated a greater tissue vascularizing effect with the SIS constructs. We surmise that in vitro conditions described in the Lin et al. study do not adequately mimic our in vivo bladder conditions and hence the data is not directly translatable. The inflammation eliciting characteristics of SIS may have also blunted vessel regeneration due to a vigorous innate immune response as previously described^17^. Finally, CD34 + HSPCs are a heterogeneous population of cells and contain subpopulations of pro-endothelial progenitor and precursor cells. As the exact number of these cells vary with each BM aspiration, it may be difficult to gauge their true effect on the tissue vascularization. Future studies may include the further dissection of the CD34 + HSPC population into pro-angiogenic or pro-vasculogenic cells to better understand the role of these cells in a bladder tissue regenerative setting.

An objective of augmentation enterocystoplasty is to restore or increase the volumetric capacity of dysfunctional bladders in order to prevent the build-up of internal bladder pressure and subsequent upper urinary tract damage. The reduced bladder capacity of SIS grafted animals following bladder augmentation may be attributed to the mechanical features of SIS. Although SIS is a durable, flexible, viscoelastic material and has been utilized successfully for multiple clinical applications^41,44–47^, it lacks structural resilience and can undergo significant graft deformation in vivo, especially in situations where graft size is small (0.5 cm × 0.5 cm × 0.01 cm) as in our bladder augmentation model. Other groups have experienced graft contraction utilizing SIS in non-urological settings^40^. As the degree of potential graft contracture for SIS was not enumerated during our studies, it is believed that the reduction in graft area that we experienced may have negatively impacted the extent at which bladder tissue regenerated, ultimately effecting bladder capacity.

Bone marrow derived MSCs and CD34 + HSPCs have been extensively studied over the last several decades and are currently the focus of multiple clinical trials in a variety of different clinical settings^48^. Along these lines, MSCs and CD34 + HSPCs have also demonstrated their regenerative prowess as viable alternative cell sources that enhance bladder tissue vascularization while simultaneously promoting overall bladder tissue regeneration^28^. The results of this study indicate that there is limited significant enhancement with regards to tissue vascularization and related bladder physiology at the 10 week time-point. This may be attributed to several factors including the inability of the model to capture the subtle nuances of increasing cell densities and the modest physical dimensions of the graft. Grafts mimicking human bladder tissue in size may provide a more reasonable approximation of cell seeding and ensuing bladder tissue regeneration. Finally, the use of this specific stem cell/POC scaffold platform may not be suitable for all patients that possess an end-stage, dysfunctional bladder and may cause fistula formation, induce bladder adhesions as well as lesions of the urothelium. Patients that exhibit severe hematuria, for example, may not be suitable candidates for this procedure. Furthermore, patients afflicted with developmental defects such as spina bifida or those undergoing radiation treatment for urological cancers may be suitable candidates for this treatment when this treatment option becomes a translational reality.

## Supplementary Information

Supplementary Information.
