# Supplementary material for: The effects of bone marrow stem and progenitor cell seeding on urinary bladder tissue regeneration
Source: Sci Rep. 2021 Jan 27;11:2322. doi: 10.1038/s41598-021-81939-5 (PMC7840904; doi:10.1038/s41598-021-81939-5)
Supplement: Supplementary file 1 — Supplementary Information. [file 41598_2021_81939_MOESM1_ESM.pdf]

The Effects of Bone Marrow Stem and Progenitor Cell Seeding on Urinary Bladder Tissue Regeneration

Matthew I. Bury, Natalie J. Fuller, Renea M. Sturm, Rebecca Rabizadeh, Bonnie G. Nolan, Milica Barac, Sonia S. Edassery, Yvonne Y. Chan, Arun K. Sharma

Supplemental Information

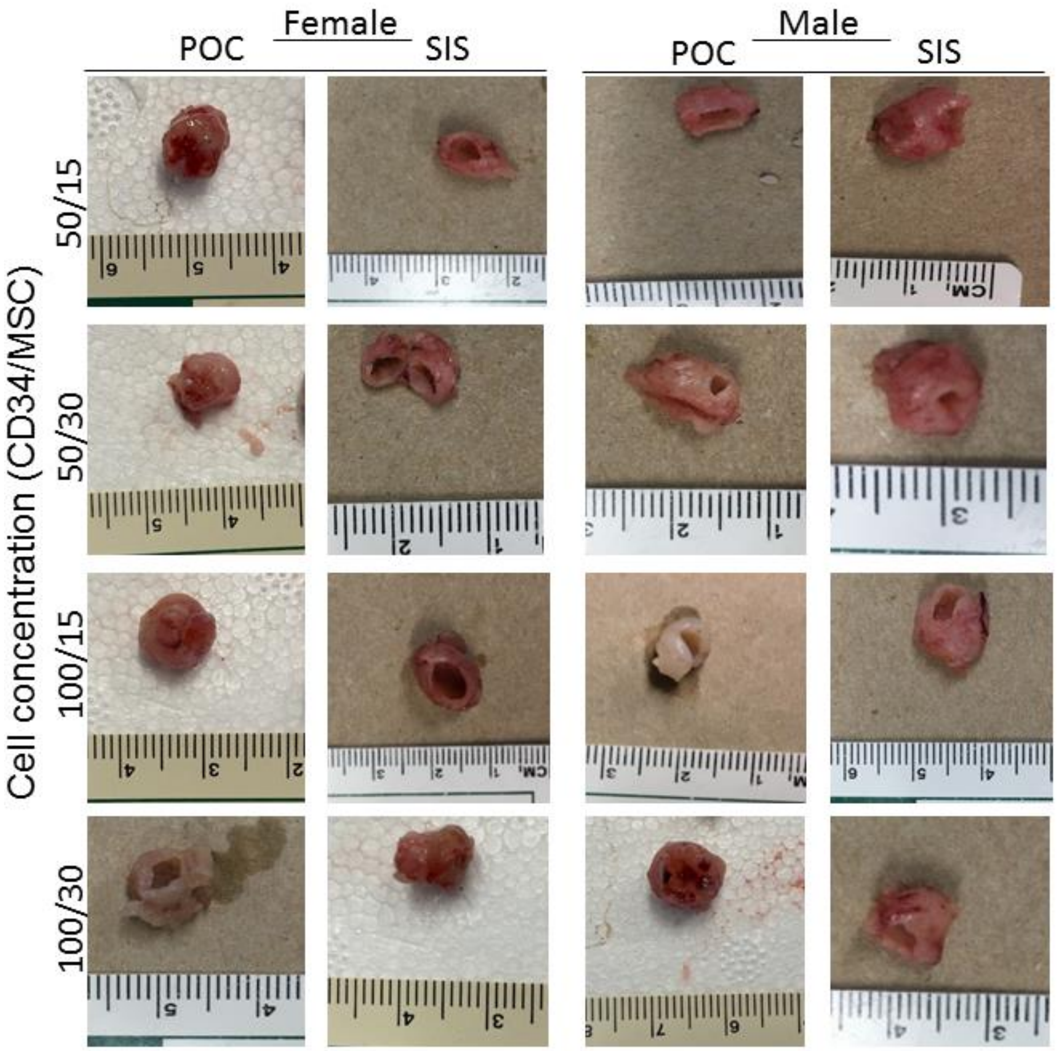

Figure S1

Rat urinary bladders 10 weeks post-augmentation. Gross images of whole rat urinary bladders that were augmented with either the POC or SIS scaffolds seeded at varying MSC and CD34+ HSPC numbers. The y-axis represents cell seeding density as follows: 50/15=50,000 CD34+ HSPCs/15,000 MSCs; 50/30=50,000 CD34+ HSPCs/30,000 MSCs; 100/15=100,000 CD34+ HSPCs/15,000 MSCs; 100/30=100,000 CD34+ HSPCs/30,000 MSCs.
